# Study reporting guidelines: How valid are they?

**DOI:** 10.1016/j.conctc.2019.100343

**Published:** 2019-03-11

**Authors:** Catherine Arundel, Sophie James, Matthew Northgraves, Alison Booth

**Affiliations:** York Trials Unit, Department of Health Sciences, University of York, York, UK

**Keywords:** Reporting guidelines, Validity, Validation

## Abstract

Reporting guidelines help improve the reporting of specific study designs, and clear guidance on the best approaches for developing guidelines is available. The methodological strength, or validation of guidelines is however unclear. This article explores what validation of reporting guidelines might involve, and whether this has been conducted for key reporting guidelines.

## 1 Introduction

Comprehensive reporting can reduce reporting bias, enable informed decision making in clinical practice, limit duplication of effort and inform subsequent research [1]. The quality of reporting of research activity continues to be inadequate, presenting readers with difficulties in judging the reliability of research findings, or how best to interpret results for individual settings [2,3].

Reporting guidelines have been developed to help improve the reporting of specific study designs. If followed by authors this should enable users to understand the design, conduct and analysis of the research, to critically appraise and review the findings and interpret the conclusions appropriately [4].

A guideline is a checklist, diagram or explicit text which guides authors in reporting research, and should be developed using explicit methodology [2]. Many already exist, mostly as checklists, and clear guidance, including a checklist of recommended steps, for developing such tools is available [2].

## 2 Use of guidelines

A search of the websites of five leading medical and health research journals (BMJ, Journal of the American Medical Association (JAMA), Lancet, New England Journal of Medicine (NEJM) and BMC Trials) identified the reporting guidelines included in the journals’ instructions to authors. These journals were purposively sampled as they: are prominent in the publication of a wide range of research topics and study designs; each publish significant volumes of research over a 3-month period (RCT publication rate, range 1–10 per month; 4–31 per quarter); and represent a range of impact factors (Range: 2.067 to 79.258).

All five journals require the use of the CONSORT reporting guidelines for randomised controlled trial (RCT) manuscripts. For RCTs, the BMJ also specifically recommend use of the TIDieR checklist to ensure accurate and complete reporting of a trial intervention [5]. BMC Trials, BMJ, JAMA and Lancet promote the use of reporting guidelines for other study designs and refer authors to the EQUATOR (Enhancing the QUAlity and Transparency Of health Research) Network database of reporting guidelines [6].

The EQUATOR Network is an international multidisciplinary group that promotes transparent and accurate health research reporting through use of reporting guidelines. The network provides access to a comprehensive range of such guidelines in a searchable database (http://www.equator-network.org). However, there is evidence that simply having reporting guidelines, even with journal endorsement of their use, is insufficient [7].

The EQUATOR Network takes an inclusive approach and are clear that there is no indication of methodological strength, or validation of the guidelines listed. The reporting guidelines for the main study types, such as CONSORT for RCTs, STROBE for observational studies, and STARD for diagnostic/prognostic studies, are highlighted on the front page of the EQUATOR Network website. We decided to explore what validation of reporting guidelines might involve.

## 3 Validation

Validation is ‘the action of checking or proving the validity or accuracy of something’ [8] principles already well established in the development of health care and research documentation.

In the UK the National Institute for Health and Care Excellence (NICE) requires validation of guidelines designed to inform decisions in health, public health and social care (https://www.nice.org.uk/about/what-we-do/our-programmes/nice-guidance). As a minimum, validation comprises stakeholder review, with fieldwork to trial implementation and discussions with service users, with external review also included if warranted, for example for guidelines in complex or sensitive areas (https://www.nice.org.uk/process/pmg20/chapter/the-validation-process-for-draft-guidelines-and-dealing-with-stakeholder-comments).

A number of initiatives provide standards for the development and validation of patient reported outcome measures (PROMs) for research [[9], [10], [11]]. This includes identifying the scope and focus of the measure, reviewing literature, engaging relevant stakeholders, and consensus assessment. Guidance for the development of health research reporting guidelines suggests similar features to those used for PROMs: 1) Literature review; 2) Delphi process; 3) Identification of key items; 4) Meeting with collaborators; 5) Iterative revision and review; 6) Pilot testing, all of which are derived from the authors’ comprehensive experience in the development of reporting guidelines [2]. While there are many similarities in the proposed activities for the development of PROMs and reporting guidelines, unlike PROMS, methods of validation of reporting guidelines are not explicitly mentioned.

In 2016 we conducted a systematic literature search using MEDLINE to identify validation methods commonly used for PROMs. Search strategies are provided in Supplementary Document 1. Our pre-defined inclusion criteria were for studies: focused on PROMs; detailing a validation method; references other publications regarding validation. Two authors independently screened the search results against the inclusion criteria, identified 73 relevant papers. Details of the included papers are provided in Supplementary Document 2. Data on PROM type, validation method, and if this was noted as a strength or limitation were extracted and a summary of the validation methods identified is detailed in Table 1, Table 2.

**Table 1:** Types of validation method used for PROMS development.

Validation Method	Number of studies using method^a^	Number of studies using single method only
Comparison to similar measures	n = 23	n = 5
Delphi consensus	n = 1	n = 1
Expert opinion	n = 1	n = 0
Focus groups	n = 3	n = 0
Interviews	n = 7	n = 3
Statistical testing	n = 56	n = 31

a Note some studies used multiple methods, therefore total n exceeds number of papers included.

**Table 2:** Combinations of validation methods used for PROMS development.

Validation Method	Number of studies using method
Statistical testing plus comparison to similar measures	n = 17
Statistical testing plus expert opinion	n = 1
Statistical testing plus focus groups	n = 1
Statistical testing plus interviews	n = 2
Statistical testing plus comparison to similar measures plus focus groups	n = 1
Statistical testing plus comparison to similar measures plus interviews	n = 1
Statistical testing plus focus groups plus interviews	n = 1

By far the most common method of validation was use of statistical testing either as a single validation method or in combination with other methods. The most common combination was statistical testing in conjunction with comparison with similar measures. This corresponds to guidance published in 2011 which indicates that comparison and correlation with similar, existing measures is critical in the development of PROMS [10].

## 4 Are reporting guidelines validated?

Having established the methods of validation, we went on to see which had been used in the reporting guidelines highlighted on the EQUATOR Network homepage.

We conducted a literature search in 2018 to identify papers reporting the development of guidelines for the main study types as highlighted on the EQUATOR network website. Two researchers independently extracted information about the development and validation methods reported; disagreements were resolved through discussion. We excluded papers where content analysis was the sole measure used, as this was not explicitly identified as a validation activity. The results are summarised in Table 3.

**Table 3:** Validation methods used in reporting guidelines for main study types.

Research Type	Key Guideline	Validation Methods
RCT	CONSORT [12]	- Stakeholder meeting - Evidence synthesis - Stakeholder review of draft
Observational Studies	STROBE [13]	- Literature search - Stakeholder meeting - Stakeholder review of drafts (following iterative review) - Peer review
Case Reports		
Systematic Review	PRISMA [14]	- Literature search - Survey of peers using systematic review methodology - Stakeholder meeting and review of drafts
Qualitative	COREQ [4]	- Literature search - Consensus discussions between authors
	ENTREQ [15]	- Literature review - Inductive generation of items - Pilot testing - Iterative review - Pilot testing
Diagnostic/Prognostic	STARD (2015) [16]	- Literature review - Survey - Stakeholder meeting - Pilot testing
	TRIPOD [17]	- Stakeholder meeting - Literature search - Survey - Stakeholder meeting and review of drafts
Economic Evaluation	CHEERS [18]	- Systematic review - Survey of collaborators - Modified Delphi panel (2 rounds)
Protocols	SPIRIT [19]	- Delphi consensus survey - Evidence synthesis - Stakeholder meetings - Pilot testing
	PRISMA-P [20]	- Mapping of existing guidelines - Stakeholder meeting and review of drafts - Delphi consensus (from PROSPERO)

The methods described within the papers matched the principles outlined in the guidance for the development of health research reporting guidelines [2]. While some guideline developers utilised multiple components and others were more selective, we believe the overarching principles remained. In the absence of clear statements, a pragmatic interpretation would say, for example, that evidence synthesis requires a literature review, and having done a literature review, or convened a stakeholder meeting, it can be supposed that validation methods were used. Of note here, is that although following the key principles, this activity is not noted as being ‘validation’. This could account for why this term is not, used in the context of promoting the use of reporting guidelines.

## 5 Discussion

Reporting guidelines, are available for a wide range of health care research methodologies [6]. Many journals request their use to increase transparent research reporting, however mandated use is rare, despite evidence that reporting guidelines can have a positive impact on completeness of reporting [7,21].

Validation is important to ensure the validity and accuracy of tools used within the conduct and reporting of research. Whilst the validation of PROMS is frequently reported, we have identified that while validation activities for reporting guidelines do occur, the activities are not always explicitly reported as such. This may occur because some validation activities are also part of the development process, for example a consensus exercise. Reporting of the development of future reporting guidelines for research, may benefit from clearly identifying the work undertaken to ensure the accuracy of the guidelines proposed. This could be within a ‘Validation’ section of a guideline publication or by simple use of the words ‘validated/validation’ in the context of the activity being reported. For completeness of reporting, it may also be appropriate to request use of a development and validation checklist, for example that provided by Moher et al. {2}, where guideline development is reported.

The EQUATOR Network database currently contains around 406 reporting guidelines which cover a variety of research methodologies, many either specialised or narrow in scope. It is unclear how many of these included validation activities in their development, and we have not been able to identify any post development or publication validation work from our literature search. Ensuring validation activities are not only undertaken but also clearly reported could add weight to the value of reporting guidelines for both those promoting their use and those authoring papers. By understanding what validation looks like, we would suggest that journals and peer reviewers could be encouraged to mandate the use of validated checklists.

Despite being included as one of the elements for the development of health research reporting guidelines, it is surprising that a limited number of the guideline development papers used pilot testing prior to publication. Given that reporting guidelines are intended to ensure transparent reporting across similar research methodologies, pilot testing may be applicable to the development of reporting guidelines.

Although we used systematic review methods to identify and select papers, we acknowledge that some may have been missed, however any impact from missed papers is likely to be limited.

## 6 Conclusion

The reporting of guidelines while including details of their development, frequently fail to explicitly identify validation activities even when they have clearly been undertaken. While this may appear to be a semantic or even pedantic issue, emphasising that reporting guidelines have been validated could help encourage authors to use the guidelines, publishers and journals to mandate checklist submission with manuscripts, and peer reviewers to monitor accuracy of completion. An improvement in any, and ideally all, of these approaches would be beneficial in promoting high quality research and reducing research waste.

## Authorship

Conception and design: CA, AB.

Analysis and interpretation of data: CA, MN, SJ.

Drafting of the article: CA.

Critical revision for important intellectual content: AB, MN, SJ.

## Conflicts of interest

CA, MN, SJ declare they have no conflicts of interest. AB is a member of the PRISMA-P group.
