# Supplementary material for: Study reporting guidelines: How valid are they?
Source: Contemp Clin Trials Commun. 2019 Mar 11;14:100343. doi: 10.1016/j.conctc.2019.100343 (PMC6421355; doi:10.1016/j.conctc.2019.100343)
Supplement: Multimedia component 1 [file mmc1.docx]

**Supplementary Document 1 – PROMS Validation Literature Search Strategy**

1. Valid$
2. Verifi$
3. Accura$
4. Precis$
5. Reliab$
6. #1 or #2 or #3 or #4 or #5
7. Test$
8. Assess$
9. Evaluat$
10. Method$
11. Process$
12. #7 or #8 or #9 or #10 or #11
13. Patient adj reported
14. person adj reported
15. #13 or #14
16. Outcome measure$ or Questionnaire$ or instrument$ or tool$ or measurement$ or scale$ or question$
17. #16 and #15
18. PROM, PROMs, PRO, PROs
19. #17 and #18
20. Outcome specific or Disease specific or Quality of life
21. health reported outcome$
22. General health or Generic health
23. #20 or #21 or #22
24. #19 and #23
25. #6 and #12 (validation and assessment)
26. #24 and #25
