# Supplementary material for: Study reporting guidelines: How valid are they?
Source: Contemp Clin Trials Commun. 2019 Mar 11;14:100343. doi: 10.1016/j.conctc.2019.100343 (PMC6421355; doi:10.1016/j.conctc.2019.100343)
Supplement: Multimedia component 2 [file mmc2.docx]

**Supplementary Document 2 – Included PROM Validation Studies**

1. Abetz L, Rajagopalan K, Mertzanis P, Begley C, Barnes R, Chalmers R, et al. Development and validation of the impact of dry eye on everyday life (IDEEL) questionnaire, a patient-reported outcomes (PRO) measure for the assessment of the burden of dry eye on patients. Health & Quality of Life Outcomes. 2011;9:111.

2. Alexis A, Daniels SR, Johnson N, Pompilus F, Burgess SM, Harper JC. Development of a new patient-reported outcome measure for facial acne: the Acne Symptom and Impact Scale (ASIS). Journal of Drugs in Dermatology: JDD. 2014;13(3):333-40.

3. Alpern AN, Brumback LC, Ratjen F, Rosenfeld M, Davis SD, Quittner AL. Initial evaluation of the Parent Cystic Fibrosis Questionnaire--Revised (CFQ-R) in infants and young children. Journal of Cystic Fibrosis. 2015;14(3):403-11.

4. Anderson RT, Baran RW, Dietz B, Kallwitz E, Erickson P, Revicki DA. Development and initial psychometric evaluation of the hepatitis C virus-patient-reported outcomes (HCV-PRO) instrument. Quality of Life Research. 2014;23(2):561-70.

5. Anderson RT, Baran RW, Erickson P, Revicki DA, Dietz B, Gooch K. Psychometric evaluation of the hepatitis C virus patient-reported outcomes (HCV-PRO) instrument: validity, responsiveness, and identification of the minimally important difference in a phase 2 clinical trial. Quality of Life Research. 2014;23(3):877-86.

6. Baumann F, Popp D, Muller K, Muller M, Schmitz P, Nerlich M, et al. Validation of a German version of the International Hip Outcome Tool 12 (iHOT12) according to the COSMIN checklist. Health & Quality of Life Outcomes. 2016;14:3.

7. Bevans M, Ross A, Cella D. Patient-Reported Outcomes Measurement Information System (PROMIS): efficient, standardized tools to measure self-reported health and quality of life. Nursing Outlook. 2014;62(5):339-45.

8. Blazeby JM, Fayers P, Conroy T, Sezer O, Ramage J, Rees M, et al. Validation of the European Organization for Research and Treatment of Cancer QLQ-LMC21 questionnaire for assessment of patient-reported outcomes during treatment of colorectal liver metastases. British Journal of Surgery. 2009;96(3):291-8.

9. Boger EJ, Hankins M, Demain SH, Latter SM. Development and psychometric evaluation of a new patient -reported outcome measure for stroke self -management: The Southampton Stroke Self - Management Questionnaire (SSSMQ). Health & Quality of Life Outcomes. 2015;13:165.

10. Bonner N, Abetz-Webb L, Renault L, Caballero T, Longhurst H, Maurer M, et al. Development and content validity testing of a patient-reported outcomes questionnaire for the assessment of hereditary angioedema in observational studies. Health & Quality of Life Outcomes. 2015;13:92.

11. Brod M, Blum SI, Bushnell DM, Ramasamy A. Development and validation of the Diabetic Peripheral Neuropathic Pain Impact (DPNPI) measure, a patient-reported outcome measure. Quality of Life Research. 2015;24(12):3001-14.

12. Brod M, Fennema H. Validation of the controlled ovarian stimulation impact measure (COSI): assessing the patient perspective. Health & Quality of Life Outcomes. 2013;11:130.

13. Brod M, Hojbjerre L, Adalsteinsson JE, Rasmussen MH. Assessing the impact of growth hormone deficiency and treatment in adults: development of a new disease-specific measure. Journal of Clinical Endocrinology & Metabolism. 2014;99(4):1204-12.

14. Brod M, Hojbjerre L, Bushnell DM, Hansen CT. Assessing the impact of non-severe hypoglycemic events and treatment in adults: development of the Treatment-Related Impact Measure-Non-severe Hypoglycemic Events (TRIM-HYPO). Quality of Life Research. 2015;24(12):2971-84.

15. Brubaker L, Piault EC, Tully SE, Evans CJ, Bavendam T, Beach J, et al. Validation study of the Self-Assessment Goal Achievement (SAGA) questionnaire for lower urinary tract symptoms. International Journal of Clinical Practice. 2013;67(4):342-50.

16. Bruce B, Fries JF. The Health Assessment Questionnaire (HAQ). Clinical & Experimental Rheumatology. 2005;23(5 Suppl 39):S14-8.

17. Canafax DM, Bhanegaonkar A, Bharmal M, Calloway M. Validation of the post sleep questionnaire for assessing subjects with restless legs syndrome: results from two double-blind, multicenter, placebo-controlled clinical trials. BMC Neurology. 2011;11:48.

18. Carrau RL, Khidr A, Gold KF, Crawley JA, Hillson EM, Koufman JA, et al. Validation of a quality-of-life instrument for laryngopharyngeal reflux. Archives of Otolaryngology -- Head & Neck Surgery. 2005;131(4):315-20.

19. Cella D, Butt Z, Kindler HL, Fuchs CS, Bray S, Barlev A, et al. Validity of the FACT Hepatobiliary (FACT-Hep) questionnaire for assessing disease-related symptoms and health-related quality of life in patients with metastatic pancreatic cancer. Quality of Life Research. 2013;22(5):1105-12.

20. Chahal J, Whelan DB, Jaglal SB, Smith P, MacDonald PB, Levy BA, et al. The multiligament quality of life questionnaire: development and evaluation of test-retest reliability and validity in patients with multiligament knee injuries. American Journal of Sports Medicine. 2014;42(12):2906-16.

21. Chua AS, Glanz BI, Guarino AJ, Cook SL, Greeke EE, Little GE, et al. Patient-reported outcomes in multiple sclerosis: Relationships among existing scales and the development of a brief measure. Multiple Sclerosis and Related Disorders. 2015;4(6):598-606.

22. Cleanthous S, Isenberg DA, Newman SP, Cano SJ. Patient Uncertainty Questionnaire-Rheumatology (PUQ-R): development and validation of a new patient-reported outcome instrument for systemic lupus erythematosus (SLE) and rheumatoid arthritis (RA) in a mixed methods study. Health & Quality of Life Outcomes. 2016;14:33.

23. Colwell HH, Mathias SD, Turner MP, Lu J, Wright N, Peeters M, et al. Psychometric evaluation of the FACT Colorectal Cancer Symptom Index (FCSI-9): reliability, validity, responsiveness, and clinical meaningfulness. Oncologist. 2010;15(3):308-16.

24. Coyne KS, Margolis MK, Thompson C, Kopp Z. Psychometric equivalence of the OAB-q in Danish, German, Polish, Swedish, and Turkish. Value in Health. 2008;11(7):1096-101.

25. Coyne KS, Tubaro A, Brubaker L, Bavendam T. Development and validation of patient-reported outcomes measures for overactive bladder: a review of concepts. Urology. 2006;68(2 Suppl):9-16.

26. Cunha-Miranda L, Santos H, Miguel C, Silva C, Barcelos F, Borges J, et al. Validation of Portuguese-translated computer touch-screen questionnaires in patients with rheumatoid arthritis and spondyloarthritis, compared with paper formats. Rheumatology International. 2015;35(12):2029-35.

27. de Souza JA, Yap BJ, Hlubocky FJ, Wroblewski K, Ratain MJ, Cella D, et al. The development of a financial toxicity patient-reported outcome in cancer: The COST measure. Cancer. 2014;120(20):3245-53.

28. Deal LS, Williams VS, Fehnel SE. Development of an electronic daily uterine fibroid symptom diary. The Patient: Patient-Centered Outcomes Research. 2011;4(1):31-44.

29. Dean K, Jenkinson C, Wilcock G, Walker Z. The development and validation of a patient-reported quality of life measure for people with mild cognitive impairment. International Psychogeriatrics. 2014;26(3):487-97.

30. Diesinger Y, Jenny JY. Validation of the French version of two on high-activity knee questionnaires. Orthopaedics & traumatology, surgery & research. 2014;100(5):535-8.

31. Dueck AC, Mendoza TR, Mitchell SA, Reeve BB, Castro KM, Rogak LJ, et al. Validity and Reliability of the US National Cancer Institute's Patient-Reported Outcomes Version of the Common Terminology Criteria for Adverse Events (PRO-CTCAE).[Erratum appears in JAMA Oncol. 2016 Jan;2(1):146; PMID: 26767562]. JAMA Oncology. 2015;1(8):1051-9.

32. El Miedany Y, El Gaafary M, Youssef S, Palmer D. Towards a multidimensional patient reported outcome measures assessment: development and validation of a questionnaire for patients with ankylosing spondylitis/spondyloarthritis. Joint, Bone, Spine: Revue du Rhumatisme. 2010;77(6):575-81.

33. El Miedany Y, El Gaafary M, Youssef SS, Palmer D. Incorporating patient reported outcome measures in clinical practice: development and validation of a questionnaire for inflammatory arthritis. Clinical & Experimental Rheumatology. 2010;28(5):734-44.

34. Flynn KE, Dew MA, Lin L, Fawzy M, Graham FL, Hahn EA, et al. Reliability and construct validity of PROMIS measures for patients with heart failure who undergo heart transplant. Quality of Life Research. 2015;24(11):2591-9.

35. Gerritsen A, Jacobs M, Henselmans I, van Hattum J, Efficace F, Creemers GJ, et al. Developing a core set of patient-reported outcomes in pancreatic cancer: A Delphi survey. European Journal of Cancer. 2016;57:68-77.

36. Gorecki C, Brown JM, Cano S, Lamping DL, Briggs M, Coleman S, et al. Development and validation of a new patient-reported outcome measure for patients with pressure ulcers: the PU-QOL instrument. Health & Quality of Life Outcomes. 2013;11:95.

37. Guex JJ, Zimmet SE, Boussetta S, Nguyen C, Taieb C. Construction and validation of a patient-reported outcome dedicated to chronic venous disorders: SQOR-V (specific quality of life and outcome response - venous). Journal des Maladies Vasculaires. 2007;32(3):135-47.

38. Heald AE, Fudman EJ, Anklesaria P, Mease PJ, Team GS. Single-joint outcome measures: preliminary validation of patient-reported outcomes and physical examination. Journal of Rheumatology. 2010;37(5):1042-8.

39. Hill J, Kang S, E B, Myers H, Blackburn S, Smith S, et al. Development and initial cohort validation of the Arthritis Research UK Musculoskeletal Health Questionnaire (MSK-HQ) for use across musculoskeletal care pathways. BMJ Open. 2016;6:e012331.

40. Hill JC, Thomas E, Hill S, Foster NE, van der Windt DA. Development and Validation of the Keele Musculoskeletal Patient Reported Outcome Measure (MSK-PROM). PLoS ONE [Electronic Resource]. 2015;10(4):e0124557.

41. Judson MA, Mack M, Beaumont JL, Watt R, Barnathan ES, Victorson DE. Validation and important differences for the Sarcoidosis Assessment Tool. A new patient-reported outcome measure. American Journal of Respiratory & Critical Care Medicine. 2015;191(7):786-95.

42. Keller SD, Yang M, Treadwell MJ, Werner EM, Hassell KL. Patient reports of health outcome for adults living with sickle cell disease: development and testing of the ASCQ-Me item banks. Health & Quality of Life Outcomes. 2014;12:125.

43. Kushner JA, Lawrence HP, Shoval I, Kiss TL, Devins GM, Lee L, et al. Development and validation of a Patient-Reported Oral Mucositis Symptom (PROMS) scale. Journal (Canadian Dental Association). 2008;74(1):59.

44. Lai JS, Nowinski CJ, Zelko F, Wortman K, Burns J, Nordli DR, et al. Validation of the Neuro-QoL measurement system in children with epilepsy. Epilepsy & Behavior. 2015;46:209-14.

45. Lasch K, Joish VN, Zhu Y, Rosa K, Qiu C, Crawford B. Validation of the sleep impact scale in patients with major depressive disorder and insomnia. Current Medical Research & Opinion. 2009;25(7):1699-710.

46. Lasch KE, Hassan M, Endicott J, Piault-Luis EC, Locklear J, Fitz-Randolph M, et al. Development and content validity of a patient reported outcomes measure to assess symptoms of major depressive disorder. BMC Psychiatry. 2012;12:34.

47. Lebwohl M, Swensen AR, Nyirady J, Kim E, Gwaltney CJ, Strober BE. The Psoriasis Symptom Diary: development and content validity of a novel patient-reported outcome instrument. International Journal of Dermatology. 2014;53(6):714-22.

48. Luo Y, Yang J, Zhang Y. Development and validation of a patient-reported outcome measure for stroke patients. Health & Quality of Life Outcomes. 2015;13:53.

49. Bushnell DM, ML M, McCarrier K, Gordon K, Chiou CF, Huang X, et al. Validation of the Psoriasis Symptom Inventory (PSI), a patient-reported outcome measure to assess psoriasis symptom severity. J Dermatolog Treat. 2013;24(5):356-60.

50. Martin ML, McCarrier KP, Chiou CF, Gordon K, Kimball AB, Van Voorhees AS, et al. Early development and qualitative evidence of content validity for the Psoriasis Symptom Inventory (PSI), a patient-reported outcome measure of psoriasis symptom severity. Journal of Dermatological Treatment. 2013;24(4):255-60.

51. Martin TP, Moualed D, Paul A, Ronan N, Tysome JR, Donnelly NP, et al. The Cambridge Otology Quality of Life Questionnaire: an otology-specific patient-recorded outcome measure. A paper describing the instrument design and a report of preliminary reliability and validity. Clinical Otolaryngology. 2015;40(2):130-9.

52. Mathias SD, Chren MM, Crosby RD, Colwell HH, Yim YM, Reyes C, et al. Reliability and validity of the Advanced Basal Cell Carcinoma Index (aBCCdex). British Journal of Dermatology. 2015;173(3):713-9.

53. Matza LS, Boye KS, Yurgin N. Validation of two generic patient-reported outcome measures in patients with type 2 diabetes. Health & Quality of Life Outcomes. 2007;5:47.

54. McKenna SP, Meads DM, Doward LC, Twiss J, Pokrzywinski R, Revicki D, et al. Development and validation of the living with chronic obstructive pulmonary disease questionnaire. Quality of Life Research. 2011;20(7):1043-52.

55. Meads DM, McKenna SP, Doward LC, Pokrzywinski R, Revicki D, Hunter C, et al. Development and validation of the Asthma Life Impact Scale (ALIS). Respiratory Medicine. 2010;104(5):633-43.

56. Mok CC, Kosinski M, Ho LY, Chan KL, Jolly M. Validation of the LupusPRO in Chinese patients from Hong Kong with systemic lupus erythematosus. Arthritis care & research. 2015;67(2):297-304.

57. Mulhall JP, Goldstein I, Bushmakin AG, Cappelleri JC, Hvidsten K. Validation of the erection hardness score. Journal of Sexual Medicine. 2007;4(6):1626-34.

58. Panchapakesan V, Klassen AF, Cano SJ, Scott AM, Pusic AL. Development and psychometric evaluation of the FACE-Q Aging Appraisal Scale and Patient-Perceived Age Visual Analog Scale. Aesthetic Surgery Journal. 2013;33(8):1099-109.

59. Potter LP, Mathias SD, Raut M, Kianifard F, Tavakkol A. The OnyCOE-t questionnaire: responsiveness and clinical meaningfulness of a patient-reported outcomes questionnaire for toenail onychomycosis. Health & Quality of Life Outcomes. 2006;4:50.

60. Pusic AL, Klassen AF, Scott AM, Cano SJ. Development and psychometric evaluation of the FACE-Q satisfaction with appearance scale: a new patient-reported outcome instrument for facial aesthetics patients. Clinics in Plastic Surgery. 2013;40(2):249-60.

61. Revicki DA, Rentz AM, Luo MP, Wong RL. Psychometric characteristics of the short form 36 health survey and functional assessment of chronic illness Therapy-Fatigue subscale for patients with ankylosing spondylitis. Health & Quality of Life Outcomes. 2011;9:36.

62. Sandhu S, Killaspy H, Krotofil J, McPherson P, Harrison I, Dowling S, et al. Development and psychometric properties of the client's assessment of treatment scale for supported accommodation (CAT-SA). BMC Psychiatry. 2016;16:43.

63. Shah HA, Dritsaki M, Pink J, Petrou S. Psychometric properties of Patient Reported Outcome Measures (PROMs) in patients diagnosed with Acute Respiratory Distress Syndrome (ARDS). Health & Quality of Life Outcomes. 2016;14:15.

64. Spiegel DM, Evans RW, Gitlin M, Mayne TJ. Psychometric evaluation of the National Kidney Dialysis and Kidney Transplantation Study symptom checklist: reliability and validity. Nephrology Dialysis Transplantation. 2009;24(2):619-25.

65. Stull DE, van Hanswijck de Jonge P, Houghton K, Kocun C, Sandor DW. Development of a frequent heartburn index. Quality of Life Research. 2011;20(7):1023-34.

66. Thorborg K, Holmich P, Christensen R, Petersen J, Roos EM. The Copenhagen Hip and Groin Outcome Score (HAGOS): development and validation according to the COSMIN checklist.[Erratum appears in Br J Sports Med. 2011 Jul;45(9):742]. British Journal of Sports Medicine. 2011;45(6):478-91.

67. Valasek T, Varga PP, Szoverfi Z, Kumin M, Fairbank J, Lazary A. Reliability and validity study on the Hungarian versions of the oswestry disability index and the Quebec back pain disability scale. European Spine Journal. 2013;22(5):1010-8.

68. van Hooff ML, Spruit M, Fairbank JC, van Limbeek J, Jacobs WC. The Oswestry Disability Index (version 2.1a): validation of a Dutch language version. Spine. 2015;40(2):E83-90.

69. Walfridsson U, Arestedt K, Stromberg A. Development and validation of a new Arrhythmia-Specific questionnaire in Tachycardia and Arrhythmia (ASTA) with focus on symptom burden. Health & Quality of Life Outcomes. 2012;10:44.

70. Walmsley S, Ravey M, Graham A, Teh LS, Williams AE. Development of a patient-reported outcome measure for the foot affected by rheumatoid arthritis. Journal of Clinical Epidemiology. 2012;65(4):413-22.

71. Watt T, Hegedus L, Groenvold M, Bjorner JB, Rasmussen AK, Bonnema SJ, et al. Validity and reliability of the novel thyroid-specific quality of life questionnaire, ThyPRO. European Journal of Endocrinology. 2010;162(1):161-7.

72. Wilburn J, McKenna SP, Twiss J, Kemp K, Campbell S. Assessing quality of life in Crohn's disease: development and validation of the Crohn's Life Impact Questionnaire (CLIQ). Quality of Life Research. 2015;24(9):2279-88.

73. Wyrwich KW, Mody R, Larsen LM, Lee M, Harnam N, Revicki DA. Validation of the PAGI-SYM and PAGI-QOL among healing and maintenance of erosive esophagitis clinical trial participants. Quality of Life Research. 2010;19(4):551-64.
